# The Impact Beyond Academia: Patent Citations of the Advanced Pharmaceutical Bulletin

**DOI:** 10.34172/apb.025.45761

**Published:** 2025-06-03

**Authors:** Mihály Hegedűs, Mehdi Dadkhah, Lóránt Dénes Dávid

**Affiliations:** ^1^Department of Finance and Accounting, Tomori Pál College, Budapest, Hungary; ^2^Chamber of Hungarian Auditors, Budapest, Hungary; ^3^Department of Sustainable Tourism, Institute of Rural Development and Sustainable Economy, Hungarian University of Agriculture and Life Sciences (MATE), Gödöllő, Hungary; ^4^Department of Tourism and Hospitality, Faculty of Economics and Business, John Von Neumann University, Kecskemét, Hungary; ^5^Department of Tourism and Hospitality, Institute of Rural Development and Sustainable Economy, Hungarian University of Agriculture and Life Sciences (MATE), Gödöllő, Hungary; ^6^Savaria Department of Business Economics, Faculty of Social Sciences, Savaria University Centre, Eötvös Loránd University, Szombathely, Hungary; ^7^Department of Tourism and Hospitality, Kautz Gyula Faculty of Business and Economics, Széchenyi István University, HU9026 Győr, Hungary

**Keywords:** Technological impact, Advanced Pharmaceutical Bulletin, Patent, Sustainable development goals, Circular economy, Patent to papers citations

## Abstract

**Purpose:**

This study aims to analyze the technological impact of papers that *Advanced Pharmaceutical Bulletin* (APB) has published through patent-to-paper citations analysis.

**Methods:**

Current research uses a Scientometric approach to analyze patent citations to published papers by the APB. The Lens has been used for collecting patents that cited related papers. Some of the data analysis was conducted using the Lens analytical tool.

**Results:**

Results show that APB’s patent-to-paper citation rate is 32.39%, above the toxicology field average (6.15%) but below pharmacology (46.33%), indicating significant technological influence. APB contributes to both science and technology, attracting global inventors.

**Conclusion:**

The patent citations metric can be used to understand how a journal contributes to technological progress. However, these methods need to be standardized and promoted to understand a journal’s real value in technology contribution.

## Introduction

*Advanced Pharmaceutical Bulletin* (APB) is an indexed, reputable journal, and according to Scimago, it is in the top quarter of the Pharmacology, Toxicology and Pharmaceutics journal category. It indicates that the journal could gain a considerable number of citations from the side of academic papers that have been published by indexed journals in Scopus. Even paper-to-paper citation is an established method in academia to gain insight about the credibility of a journal and its contribution in science; patent-to-paper citation, where a patent cites academic papers, can provide valuable insights.

 The World Intellectual Property Organization (WIPO) defines a patent as “*A patent is an exclusive right granted for an invention. Patents benefit inventors by providing them with legal protection of their inventions. However, patents also benefit society by providing public access to technical information about these inventions, and thus accelerating innovation*”. A patent document describes inventive technology and also contains metadata related to the patent (i.e., inventor, owner, date, references, etc.) The analysis of patents helps companies and experts to understand new technologies and is valuable for research and development efforts.^1^ Patent documents support the United Nations Sustainable Development Goals and the circular economy by fostering innovation and providing solutions to achieve them.^2-5^ Analysis of patent-to-paper citations illuminates the impact of research beyond academia, highlighting its contributions to industry, product development, and technological innovation. Patent to paper citations show how knowledge from academia transferred to industry and added more value. This analytical method can serve as a proxy for gauging knowledge transfer to industrial practice, although it is acknowledged that such an indicator may possess inherent limitations or ‘noise’.^6^ Patents have been recognized as an indicator of technological innovation, so the patent citation analysis can be used to gain insight about the influence of science on technology where patents cite non-patent literature (i.e., papers, books, etc.). In such situations, papers cited by a patent can be considered indicative of innovative or technology-based research. However, the relationship is not straightforward: non-patent literature may include diverse materials, and only a subset of citations directly relates to the invention.^7^

 Research indicates that citation of patents to papers of a journal can be a new perspective to analyze journals in terms of practical value, rather than using traditional methods such as the impact factor.^8^ There is research that analyzed citations of patents to papers to understand the technological impact of literature.^9-11^ Therefore, the analysis of patent citations to papers offers valuable insights into a journal’s technological impact and its broader contributions to technological development. Accordingly, this editorial assesses the technological impact of APB by examining patent citations to its publications.

## Methods

 This research aims to analyze APB in terms of technological impact. In this regard, the patent citations to APB’s papers will be gathered from Lens (https://www.lens.org). The Lens offers free access to a comprehensive collection of patents and scholarly literature, supporting research and discovery across various fields. Furthermore, The Lens allows for the retrieval of citing patents and scholarly literature, facilitating their analysis.^12,13^ The dataset analyzed, obtained from The Lens, includes scholarly literature and patents without restriction to a specific publication date range. The data retrieval date was 23 April 2025. Using the analysis features available on The Lens platform, the following metrics were identified: the total number of patent citations to papers, institutions contributing to APB publications, top fields of study, contributing countries, the number of citing patents, top patent applicants, the distribution of patent documents by jurisdiction, and top CPC classification codes.

## The Results

 The query below was searched in The Lens to retrieve all indexed APB publications and identify their patent citations: *source.title: (ADVANCED PHARMACEUTICAL BULLETIN)*

 The Lens provided 1142 documents published by APB. The patent-to-paper citation rate was calculated as (number of patent citations ÷ total papers) × 100. According to the presented result, the 368 patents provided 370 citations to published papers in APB. The search for Pharmacology as the field of published papers in Lens provided 2 076 267 scholarly works that received 961 959 citations from 337 292 patents. This means that in pharmacology, the average number of patent-to-paper citations is about 46.33 percent. In Toxicology, 381 062 scholarly works received a total of 23 466 citations by 19 067 patents. It means that the average ratio of patent to paper citation is about 6.15%. The patent-to-paper citation rate for APB is approximately 32.39%, which is competitive compared to the average for this field.

**Figure 1:**
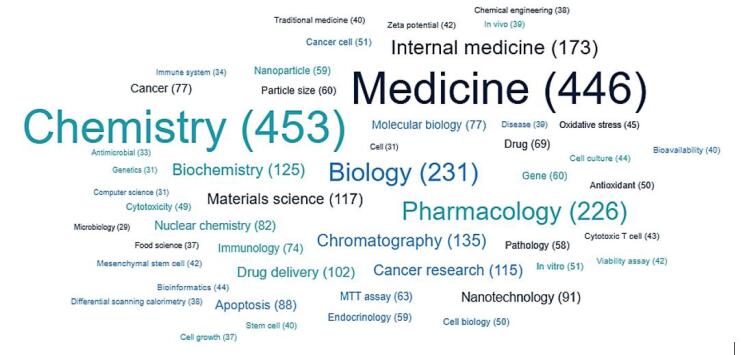
Prominent research areas published in the Advanced Pharmaceutical Bulletin (The relative prevalence of each research field is indicated by the font size of the corresponding term)

 Analysis of APB’s published papers on The Lens platform (Figure 1) identified Medicine, Chemistry, Biology, and Pharmacology as the top fields, illustrating the interdisciplinary nature of APB’s research. Separately, a search was conducted on The Lens to determine the average patent-to-paper citation rates within these general fields. The results indicate that the average patent-to-paper citation rates are approximately 10% for Medicine, 28% for Chemistry, and 43% for Biology. These figures serve as benchmarks for comparison. Thus, when compared to the average patent-to-paper citation rates in these fields, APB demonstrates a competitive ratio.

**Figure 2:**
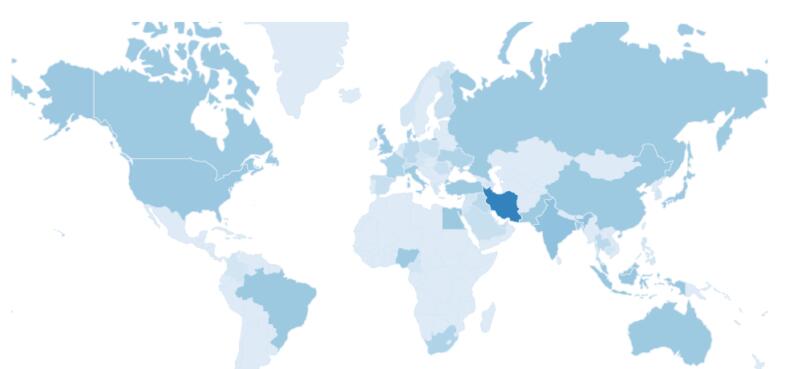
Geographical distribution of author contributions to the Advanced Pharmaceutical Bulletin by country/region (Darker colors denote a higher volume of research output)

Figure 2 shows countries where their researchers contributed to the APB publication. Even the Iran is a core country for most authors, there are international collaborations on papers that have been published by APB. The APB is popular among Iranian researchers but has also been recognized as the venue for publications with international collaboration.

**Figure 3:**
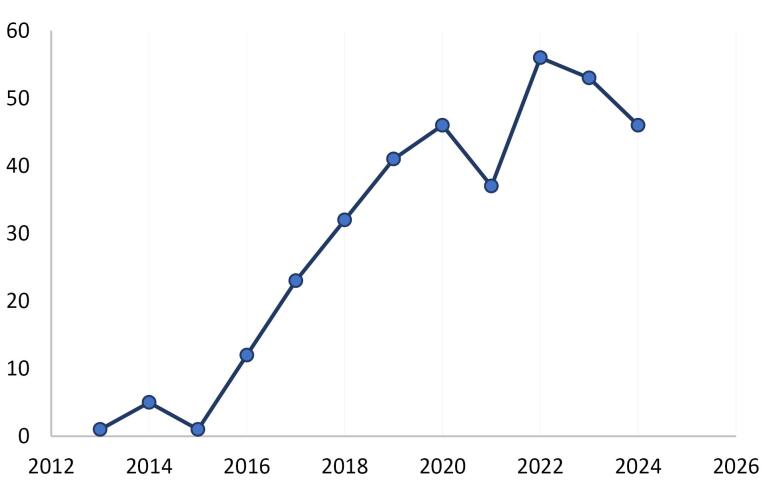
Annual count of patent documents citing Advanced Pharmaceutical Bulletin papers

**Figure 4:**
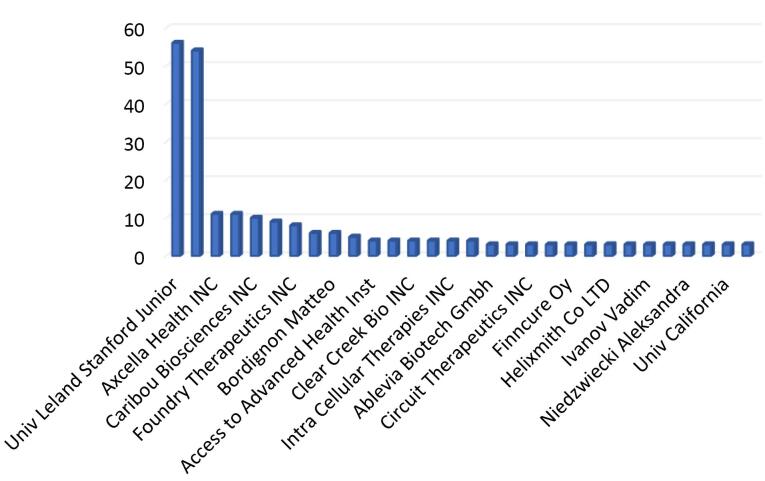
Leading patent applicants citing research from the Advanced Pharmaceutical Bulletin

Figure 3 illustrates how patents that cited APB publications are published over time. Most of the cited patents come after 2016. Figure 4 shows the top applicants. Applicant in the patent literature is an entity (individual, company, or legal entity) that submits a patent application to the patent office and aims to gain protection for an invention.^14^ Based on Figure 4, the top applicants are Univ Leland Stanford Junior (USA), Modernatx INC (USA), Axcella Health INC (USA), Deisseroth Karl (A professor in USA), Caribou Biosciences INC (USA), Arcutis Biotherapeutics INC (USA), Foundry Therapeutics INC(USA), and Bella Aurora Labs S A (Spain).

**Figure 5:**
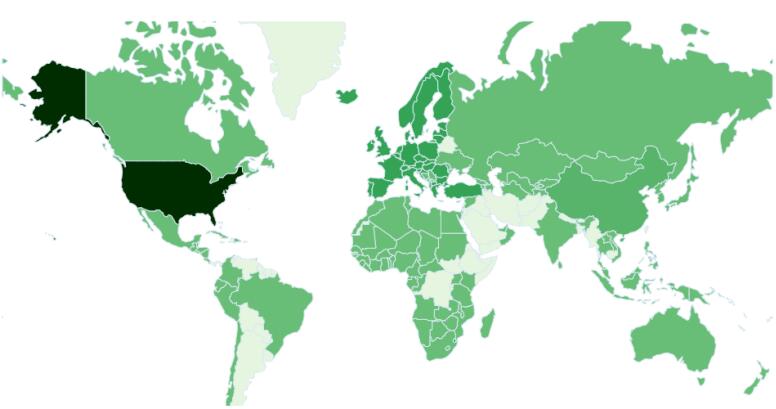
Geographical breakdown of patents citing Advanced Pharmaceutical Bulletin papers, by jurisdiction

 The patent by Jurisdiction (Figure 5) indicates that most of the patents that cited APB publications are protecting invention rights in the USA. Patent jurisdiction shows where a patent right is protected. Indeed, the protection of patents is territory-based, and the applicant should follow related guidelines to protect an invention in the desired territory.^15^

**Figure 6:**
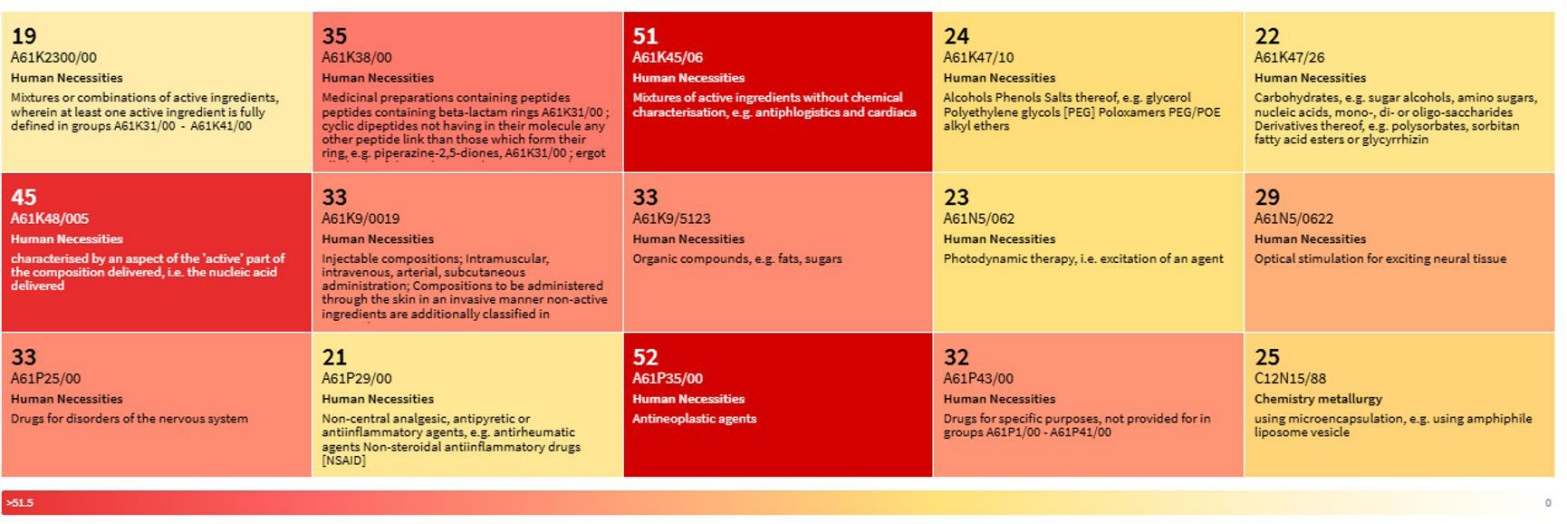
Most frequent Cooperative Patent Classification codes found in patents citing Advanced Pharmaceutical Bulletin papers

 Cooperative Patent Classification or CPC classification codes are classification themes based on technology developed by a collaboration of the European Patent Office (EPO) and the United States Patent and Trademark Office (USPTO) that facilitate patent examination, searching, and analysis globally.^15^ The top CPC of patents that cited the APB publication are shown in Figure 6. This patent set is heavily concentrated in pharmaceuticals, with major emphasis on cancer treatments, advanced drug delivery systems (especially injectables, nanoparticles/liposomes), and biologics (peptides, nucleic acids). There is also a significant focus on neurological drugs and secondary interests in pain/inflammation management and light-based therapies. The classifications suggest innovation in both the active therapeutic agents and the methods used to formulate and deliver them.

## Discussion

 The results indicate that APB is a resource for papers that provide technological impact, and could contribute to technology as a journal in the field. Even though most of the authors of a journal come from Iran, its technological impact is considerable all over the world, as most of the patents that cite APB’s papers are protected in the USA, WIPO, and European patents. An analysis of the patent inventors citing APB’s papers revealed no Iranian inventors within the top 20 most frequent contributors. Regarding patent-to-paper citations, APB demonstrates an average rate of approximately 32.39%. This figure is notably higher than the average rates observed in related disciplines and places APB within the top quartile when compared across related research fields. This indicates that APB has significantly contributed to both scientific knowledge and technological innovation, serving as a valuable outlet for researchers with novel findings and a resource for inventors and patent examiners, informing new inventions.

## Conclusion

 This paper aimed to analyze APB in terms of technological impacts by considering its citations that come from patents. The results indicate that APB has a good history in publishing technology-based papers and could receive a fair number of patent-to-paper citations in comparison to peers in the field. The published papers in APB could attract inventors from all over the world, even individuals from standout medical companies. This journal is a valuable source for authors, inventors, and examiners in terms of publishing innovative research or finding innovative ideas to use in inventions.

 For future research, it is recommended that a metric that could analyze the technological impact of a journal be standard for academic institutions to analyze the technological value of journals and publish results globally.

 Due to differences in the coverage of patent and academic databases, the use of various platforms such as Scopus or SciVal may yield different numerical values for the patent-to-paper citation rate. Patent databases often vary in the extent of their patent office data coverage, while academic databases differ in their criteria for indexing scholarly publications. However, these discrepancies do not undermine the overall conclusion that APB can have an impact beyond academia.

## Competing Interests

 None declared.

## Ethical Approval

 Not applicable.
